# Species identification through deep learning and geometrical morphology in oaks (*Quercus* spp.): Pros and cons

**DOI:** 10.1002/ece3.11032

**Published:** 2024-02-13

**Authors:** Min Qi, Fang K. Du, Fei Guo, Kangquan Yin, Jijun Tang

**Affiliations:** ^1^ School of Ecology and Nature Conservation Beijing Forestry University Beijing China; ^2^ School of Computer Science and Engineering Central South University Changsha Hunan China; ^3^ School of Grassland Science Beijing Forestry University Beijing China; ^4^ Shenzhen Institute of Advanced Technology Chinese Academy of Sciences Shenzhen Guangdong China

**Keywords:** deep learning, genetic assignment, geometric morphometrics, leaf shape

## Abstract

Plant phenotypic characteristics, especially leaf morphology of leaves, are an important indicator for species identification. However, leaf shape can be extraordinarily complex in some species, such as oaks. The great variation in leaf morphology and difficulty of species identification in oaks have attracted the attention of scientists since Charles Darwin. Recent advances in discrimination technology have provided opportunities to understand leaf morphology variation in oaks. Here, we aimed to compare the accuracy and efficiency of species identification in two closely related deciduous oaks by geometric morphometric method (GMM) and deep learning using preliminary identification of simple sequence repeats (nSSRs) as *a prior*. A total of 538 Asian deciduous oak trees, 16 *Q. aliena* and 23 *Q. dentata* populations, were firstly assigned by nSSRs Bayesian clustering analysis to one of the two species or admixture and this grouping served as a priori identification of these trees. Then we analyzed the shapes of 2328 leaves from the 538 trees in terms of 13 characters (landmarks) by GMM. Finally, we trained and classified 2221 leaf‐scanned images with Xception architecture using deep learning. The two species can be identified by GMM and deep learning using genetic analysis as a priori. Deep learning is the most cost‐efficient method in terms of time‐consuming, while GMM can confirm the admixture individuals' leaf shape. These various methods provide high classification accuracy, highlight the application in plant classification research, and are ready to be applied to other morphology analysis.

## Graphical abstract

We conducted a comparative analysis to evaluate the accuracy and efficiency of the GMMs and deep learning methods in discriminating two closely related oaks, *Q. aliena* and *Q. dentata*, using genetics as a priori classification. We found that deep learning is the most cost‐efficient method in terms of time and cost, whereas GMMs can confirm the leaf shape of admixture individuals and demonstrate the tendency of leaf contraction and expansion. Moreover, we found that the shape of admixture individuals was close to that of *Q. dentata*, suggesting that oaks retain high levels of fitness variation, with *Q. aliena* being more favored by selection in leaf morphological traits.

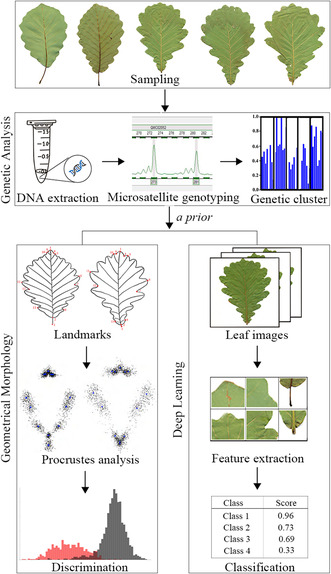

## 1 BACKGROUND

Accurate species identification is a key prerequisite for ecological, evolutionary, and conservation studies (Cope et al., 2012; Wang et al., 2022). Phenotypic characteristics such as leaf shape are the most intuitive and effective indicators for species identification. Leaf shape, the core of taxonomy and systematics, is recognized as a trait with great functional significance (Nicotra et al., 2011). However, plant identification by leaf shape can be challenging because of natural hybridization, introgression, and incomplete lineage sorting (Darwin, 1872; Rieseberg et al., 2006). Fortunately, recent advances in discrimination technology have provided opportunities to understand leaf shape variations. However, a detailed comparison of the accuracy and efficiency of this species identification method is lacking.

Traditional morphological methods of leaf shape measurement of quantitative and qualitative variables such as distances, angles, areas, and number of veins can be effectively for species identification (Henderson, 2006; Kremer et al., 2002; Marcus, 1990). However, such variables often do not share common units and comparable ranges of variation, and identification results are frequently affected by leaf size and are unlikely to intuitively express leaf shape variation in an interpretable figure (Mitteroecker & Gunz, 2009). To solve this problem, geometric morphometric method (GMM) digitizes the original geometry of the leaf shape based on the Cartesian coordinates of landmarks and generates quantitative descriptions of leaf shape (Klingenberg, 2011; Ray, 1992; Zelditch et al., 2004). The multivariate statistics of GMM can visualize leaf shape variation by translation, scaling, and rotation, regardless of the leaf location, direction, and size, making the results more intuitive and efficient (Mitteroecker & Gunz, 2009; Viscosi & Cardini, 2011). In particular, there is documentary evidence of generating quantitative descriptions of leaf shape, and these have been found to be quite effective for comparing shapes within and among species (Du et al., 2022; Li et al., 2021; Liu et al., 2018; Viscosi et al., 2009). However, the above method assumes some known shape attributes or landmarks and might miss small interactive effect (Fu et al., 2017).

Modern molecular techniques provide another method for species identification by classifying individuals in pure or mixed genotypes without *priori* information (Guichoux et al., 2011; Pritchard et al., 2000). Microsatellite analysis has frequently been used to assess the frequency of alleles between species under the assumption that species taxonomy is unknown (Agarwal et al., 2008; Guichoux et al., 2011). In particular, when using morphological characteristics for species classification, a priori grouping using microsatellite molecular approaches could provide a more reliable identification of species.

With the rapid development of machine learning, image‐based deep learning methods have been increasingly applied in the field of plant recognition using machine self‐learning to identify key features from massive image data (Hinton & Salakhutdinov, 2006; Pawara et al., 2017; Sun et al., 2017). The wide application of deep learning is based on the rapid development of multilayered neural networks with three main parts: an input layer, a hidden layer (the processing core), and an output layer, which provides a toolbox for high‐dimensional data (Olden et al., 2008). Convolutional neural network (CNN) was introduced by LeCun et al. (1989) as a supervised feedforward neural network algorithm. Owing to its ease of training and generalization, CNN has become a common neural network for image processing (LeCun et al., 2015). CNN has been widely used in various fields, including target detection (He et al., 2015), and speech recognition (Hinton et al., 2012), and made remarkable contributions to the application of image classification (Liu et al., 2019). In 2014, GoogLeNet (Inception V1) won the championship at ImageNet large‐scale visual recognition challenge (ILSVRC), later refined as Inception V2 and Inception V3 (Ioffe & Szegedy, 2015; Szegedy, Liu, et al., 2015; Szegedy, Vanhoucke, et al., 2015). The Xception is an improved version underlying the Inception architecture, standing for “Extreme Inception” (Chollet, 2017). The Xception architecture is a linear stack of deeply separable convolutional layers with residual connections. A depth‐wise separable convolution can be understood as an inception module with a maximally large number of towers. It is a novel deep convolutional neural network architecture inspired by inception that performs well on the ImageNet dataset (Chollet, 2017). Experimental evaluation of the Xception model found that the top‐5 accuracy of the Xception for classification on the ImageNet database was 94.5% (Chollet, 2017). Compared with previous traditional machine learning algorithms, image acquisition can quickly convert plant morphological information into abstract feature maps by deep learning without human supervision, greatly simplifying the process of plant phenotypic data acquisition (Christin et al., 2019).

*Quercus* L. (oaks) is one of the most diverse and ecologically important tree genera in Northern Hemisphere, with high species diversity in North America and South‐East Asia (Denk et al., 2018). High frequency of natural hybridization and introgression confound the interspecific boundary, making oak species identification extremely complex (Darwin, 1872; Gerber et al., 2014; Manos et al., 1999; Rieseberg et al., 2006). In addition, wide geographic distribution and a variety of environmental conditions strongly influence leaf variation, making morphological characteristics alone weak for distinguishing oak species (Maya‐García et al., 2020; Nagamitsu et al., 2020). Therefore, oaks are considered to be classic models for species identification (Viscosi et al., 2011).

In this study, we selected two closely related Asian white oak species, *Quercus aliena* Blume and *Quercus dentata* Thunberg, which belong to a small monophyletic group of oak species (Hipp et al., 2020; Hubert et al., 2014). *Q. aliena* and *Q. dentata* are the main forest tree species making up the mountainous vegetation areas of East Asia. They have a wide geographic distribution, mainly distributed on sunny slopes with an altitude range of 100–2000 meters and often co‐occur side by side in some forests (Huang et al., 1999). Previous studies showed that both species can be discriminated by leaf shape (Du et al., 2022; Liu et al., 2018). However, a single method is not sufficient to identify species. Combining the evidence of morphological, molecular, and deep learning can effectively improve the classification of individuals and result in a higher resolution of species delimitation (Beatty et al., 2016; Rellstab et al., 2016). In this study, we systematically sampled individual trees of *Q. aliena* and *Q. dentata* distributed in China and used GMM and deep learning to answer the following questions: (1) Can the two related species be identified through GMM and deep learning based on a priori identification by genetics? (2) What is the accuracy and efficiency of the above approaches for species identification? (3) Their potential application in other morphology analysis.

## 2 MATERIALS AND METHODS

### 2.1 Genotypic and morphological data

**FIGURE 1:**
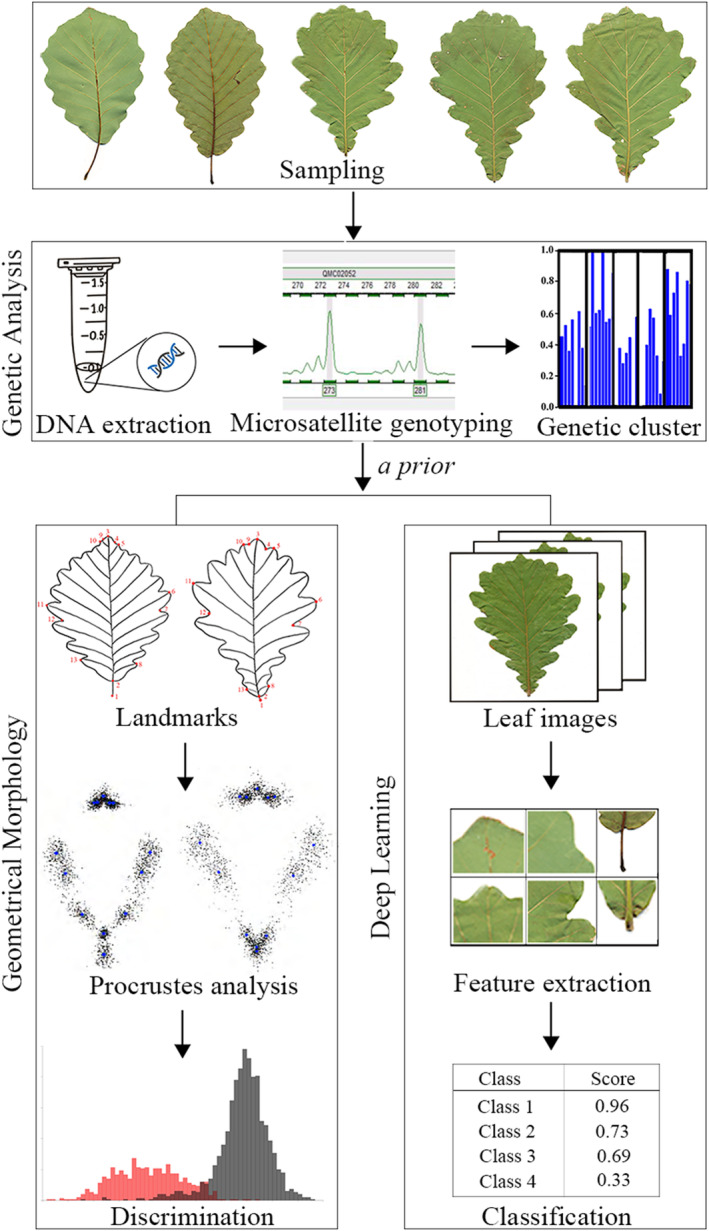
Strategy for leaf shape identification by geometric morphometric method (GMM) and deep learning with the genetic analysis served as a priori classification.

As ecological character displacement (ECD) might occur in its sympatric distribution we deliberately excluded the co‐occurring sites in this study because the discrimination rate in sympatry is higher than 92% by morphology assignment for the species pair (Du et al., 2022). In short, we conducted a random sampling of 538 individuals from 39 natural oak populations spaced over 30 km apart, including 16 *Q. aliena* populations and 23 *Q. dentata* populations, covering nearly the entire distribution in China (Figure S1, Table S1). For each population, we collected three to six fully developed and mature leaves from each individual along the four cardinal directions in the middle layer of the canopy, at least 10 m apart. Genotypic data of the 538 individuals using 12 nuclear microsatellite loci and four fluorescent dyes were from Du et al. (2022). Loci with non‐overlapping allele size ranges were labeled with the same fluorescent dye, whereas those with overlapping allele size ranges were labeled with different dyes and resolved individually because of the different characteristic emission spectra of each dye. Morphological data of 2328 leaves with 13 landmarks were from Du et al. (2022) (Figure 1, Table S3). These landmarks were converted to 13 pairs of Cartesian coordinates (*x*, *y*) as raw input data for morphological analysis (Klingenberg, 2011).

### 2.2 Model‐based clustering using genetic data

We employed Bayesian cluster analysis to assign individuals to *K* clusters without any species identification information using structure v. 2.3.4 (Pritchard et al., 2000). The program was implemented in 200,000 Markov Chain Monte Carlo cycles (MCMC), following 100,000 burn‐in cycles. We performed 20 iterations for each *K* value ranging from 1 to 10. To determine the most likely number of clusters, we used Pr(*X*|*K*) and Δ*K* implemented in the structure harvester program (Earl & VonHoldt, 2012; Evanno et al., 2005). We then used an admixture coefficient (*Q*) value to define whether the sampled individuals were purebreds or admixtures with a threshold value of 0.9, based on previous work in oaks (Lepais et al., 2009; Liu et al., 2018; Peñaloza‐Ramírez et al., 2010; Viscosi et al., 2012). This dataset served as a priori classification for *Q. aliena* and *Q. dentata*. In addition, we performed a principal coordinate analysis (PCoA) based on the genetic distance matrix using genalex v. 6.5 (Peakall & Smouse, 2012) and displayed the distribution frequency of principal component (PC) scores for all individuals to visualize the individuals' genetic proximities using the “vegan” package in R (Oksanen et al., 2022).

### 2.3 Multivariate analyses of leaf morphology

We first performed a generalized procrustes analysis (GPA) to minimize the difference between the corresponding landmarks by translation, scaling, and rotation using the morphoj program (Figure 1) (Klingenberg, 2011; Rohlf & Slice, 1990). Five outliers that significantly deviated from the average configuration were excluded as default setting. We created a wireframe, and sets of lines linking the landmarks in a configuration, that can be used to visualize shape changes. Finally, we generated a covariance matrix of the average configuration at the leaf‐level for the leaf shape variation analysis (Viscosi et al., 2009).

To visualize the differences in leaf shape between species, we conducted two distinct multivariate statistical analyses using the morphoj program, utilizing the genetic delimitation of *Q. aliena* and *Q. dentata* individuals as grouping variables for species discrimination. The first analysis employed canonical variate analysis (CVA), while the second employed discriminant analysis (DA) (Klingenberg, 2011). These two methods aim to combine the original variables into independent composite variables that explain the largest part of the total variation in leaf shape. CVA maximizes the separation of specified groups based on Procrustes and Mahalanobis distances with permutation tests (*T* ^2^ statistics; 10,000 permutations per test) to investigate three or more groups. DA mainly focuses on the difference between two groups through cross‐validation scores classification with *T* ^2^ statistics (*p* value for tests with 1000 permutations <.0001; Klingenberg, 2011).

### 2.4 Deep learning discrimination based on image recognition

**FIGURE 2:**
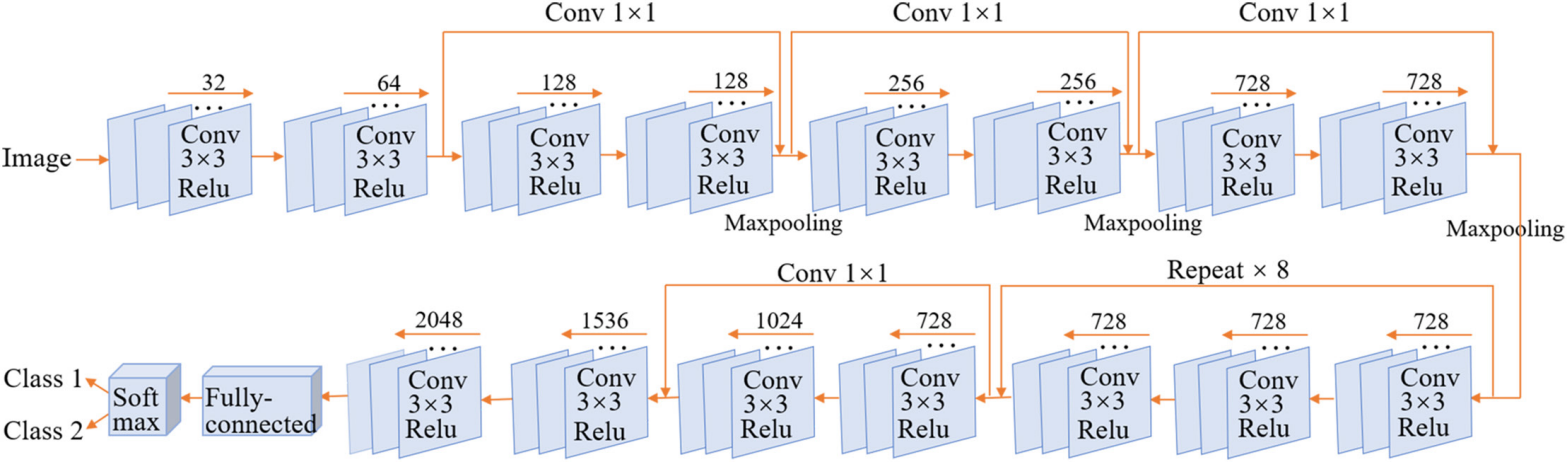
The schema of Xception model with 36 convolutional layers.

We used a total of 2221 scanning images for deep learning classification comprising 539 *Q. aliena* images, 1202 *Q. dentata* images, and 480 admixture images determined through genotyping (Figure 1). To achieve clear classification, we manually divided all images into the following four data sets: *Q. aliena* (539 images) vs. *Q. dentata* (1202 images), *Q. aliena* (539 images) vs. admixture (480 images), *Q. dentata* (1202 images) vs. admixture (480 images), and *Q. aliena* (539 images) vs. *Q. dentata* (1202 images) vs. admixture (480 images). We randomly divided each data set into three subsets for training, validation, and testing in the proportion of 70: 15: 15. We then used the Xception architecture with 36 convolutional layers to form the feature extraction base of the network. A rectified linear unit was used as the activation function (Figure 2) (Nair & Hinton, 2010). We selected SoftMax function as the classifier. The training, verification, and testing data sets were implemented on NVIDIA Tesla K80 GPUs using the TensorFlow 2.0 framework (Abadi et al., 2016). We visualized testing data using t‐distributed Stochastic Neighbor Embedding (t‐SNE) tools by giving each datapoint a location on a two‐dimensional map (Van der Maaten & Hinton, 2008).

Optimal model parameters (convolutional kernel and batch normalization) were selected using the training data and applied to the test data set for estimating prediction performance. Different indicators are obtained to evaluate the classification results. The classification accuracy rate (predicted value/true value) was calculated as follows:

$$\text{Accuracy}=\frac{\mathrm{TP}+\mathrm{TN}}{\mathrm{TP}+\mathrm{TN}+\mathrm{FP}+\mathrm{FN}}$$

Here, the true positive rate (TP) indicates accurate positive identifications are correctly predicted, and true negative rate (TN) indicates accurate negative identifications are correctly predicted. False negative rate (FN) indicates that a true observation is predicted to be different. False positive rate (FP) indicates that the observation is different but predicted as true. Additionally, it is important to consider more detailed parameters:

$$\text{Recall}=\frac{\mathrm{TP}}{\mathrm{TP}+\mathrm{FN}}$$

$$\text{Precision}=\frac{\mathrm{TP}}{\mathrm{TP}+\mathrm{FP}}$$

A high recall indicates that the species is correctly recognized (a small number of FN). A high Precision indicates that an example labeled as positive is indeed positive (a small number of FP). Although there is no necessary correlation between precision and recall based on the calculation formula, they are often interdependent in large‐scale data sets. Therefore, it is necessary to consider both parameters equally. The F‐score, calculated as the harmonic mean of precision and recall, provides a comprehensive measure (Narkhede, 2018):

$$F-\text{score}=\frac{2*\text{Precision}*\text{Recall}}{\text{Precision}+\text{Recall}}$$

### 2.5 Effectiveness measurement of different methods

We compared and quantified the efficiency of different approaches for species identification using a time‐effectiveness and cost‐effectiveness metric by converting the cost of experimental consumables and labor. Taking a sample size of 1000 as an example: for genotyping, we required five plant genomic DNA extraction kits (Tiangen, Beijing, China) at a cost of 1000 RMB each. The estimated cost for PCR, including reagents (PCR kit, unlabeled primers, labeled primers, and labeled size standard) and consumables, was 5 RMB per reaction, with a requirement of 120 reactions. The total cost of capillary electrophoresis detection is 10,000 yuan. Thus, the total cost of the experimental consumables amounted to 17,146 RMB. For salary cost, the researchers involved in this experiment were 50 RMB/h. The total time required for DNA extraction and PCR amplification of 1000 samples was 400 h, resulting in a cost of 21,500 RMB. Consequently, the total cost of species identification by nSSR was 37,046 RMB, which is similar to the pseudo‐multiplexing SSR genotyping cost reported by Guichoux et al. (2011) despite in difference in the salary cost in different countries.

For geometric morphology, five leaves were scanned for each individual, for 10 min. Each leaf was marked with 13 landmarks and exported data, this process required 8 min for each individual (five leaves). Researchers involved in this process, without the need for a scientific background, were paid 50 RMB/h for their labor. The total time required for leaf scanning and marking 1000 samples was 300 h. Consequently, the total cost of species identification based on geometric morphology is 15,000 RMB.

For deep learning, it takes 1 min to arrange leaf pictures randomly, rename them, and clearly label the classification for each image. Researchers involved in this progress, which required background knowledge in artificial intelligence, were paid 100 RMB/h for their labor. Thus, the total cost amounted to 8333 RMB based on the calculation of (1/60) × 5 × 1000 × 100.

## 3 RESULTS

### 3.1 Genotyping assignment

**FIGURE 3:**
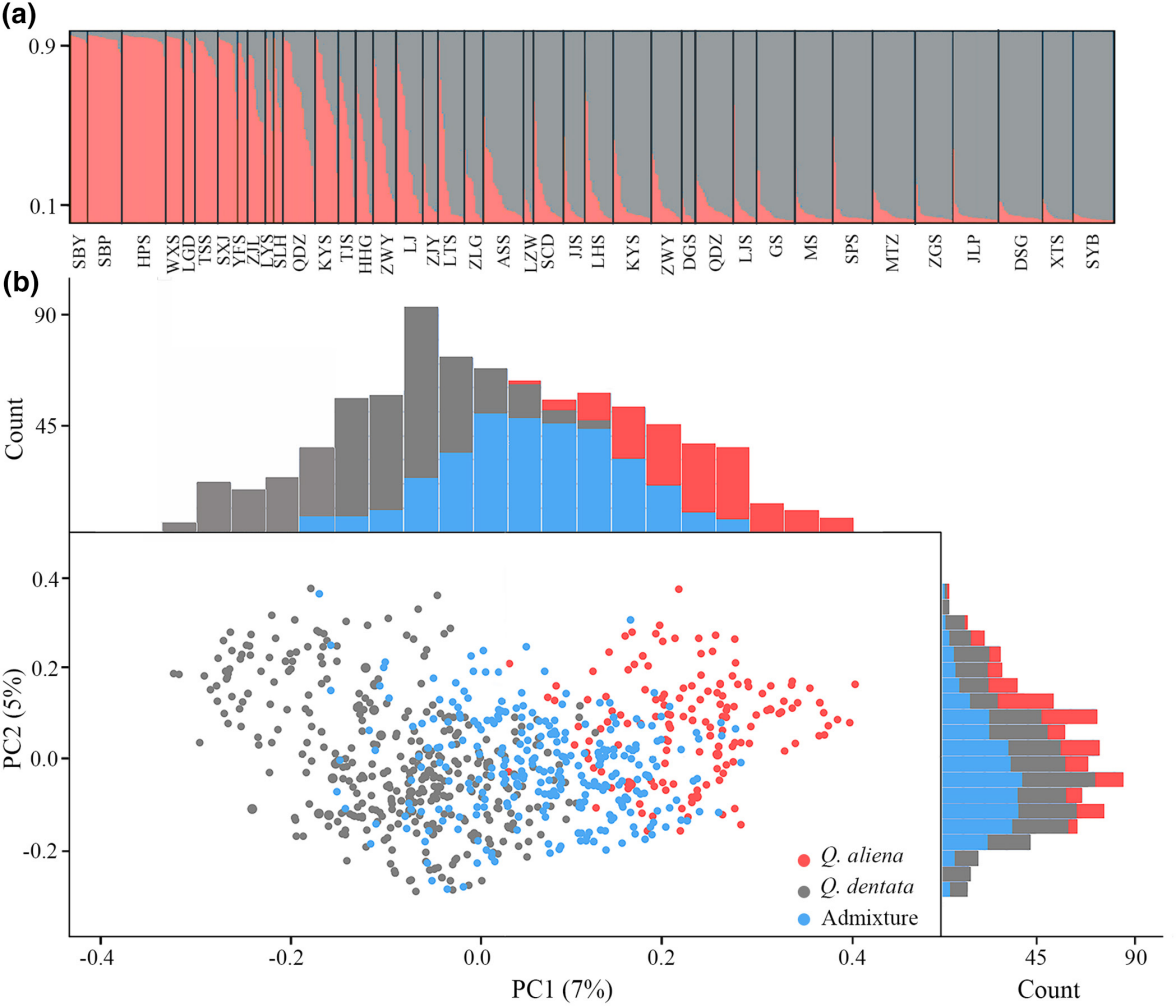
Genetic assignment and differentiation between *Quercus aliena* and *Quercus dentata*. (a) Structure analysis for *K* = 2 with different populations separated by black lines. (b) Principal component analysis (PCoA) for *Quercus aliena*, *Quercus dentata*, and admixture, with the distribution frequency of the PC1 and PC2 values plotted on the top and right sides of the scatter plot. Percentage of total variance explained by each axis is noted in brackets.

Using the Bayesian clustering method implemented in structure, we found that Delta *K* and LnP (*K*) statistics strongly suggested presence of two major clusters in the dataset (Figure 3a, Figure S2). Based on a threshold value *Q* of 0.9, we assigned 248 individuals to pure *Q. dentata* (*Q* ≤ 0.1), 132 individuals to pure *Q. aliena* (*Q* ≥ 0.9), and 158 individuals to the admixture (0.1 < *Q* < 0.9) (Figure 3a). In addition, PCoA results based on the genetic distance matrix at the individual level showed significant genetic differentiation between *Q. aliena* and *Q. dentata* with admixture intermingled, largely concordant with the structure analysis (Figure 3b). In this study, genetic data were used as a priori for both leaf morphology analysis and deep learning identification.

### 3.2 Leaf morphological variation

**FIGURE 4:**
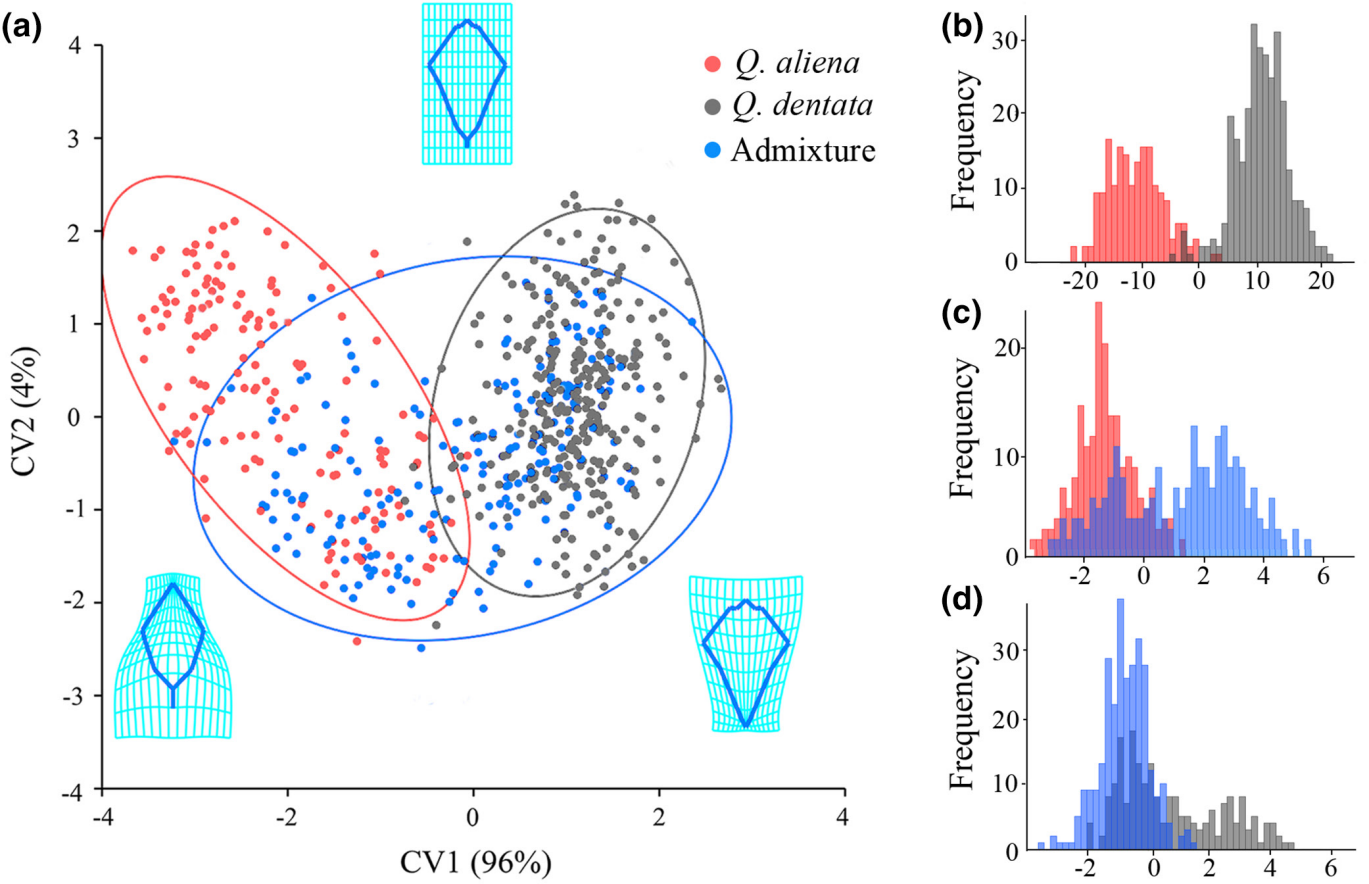
Leaf geometric morphometric analysis. (a) Scatter plot of the canonical variate analysis (CVA) at individual level with 90% confidence ellipses. Transformation grids represent the extreme leaf shape of *Quercus aliena*, *Quercus dentata*, and admixture. Discriminant analysis (DA) for the leaf shape differentiation of (b) *Quercus aliena* vs. *Quercus dentata*, (c) *Quercus aliena* vs. admixture, and (d) *Quercus dentata* vs. admixture.

The CVA score plots revealed significant morphological differences between *Q. aliena* and *Q. dentata*. Mixed individuals were scattered between the two species, closer to the *Q. dentata* cluster (Figure 4a). The transformation grids showed that the main differences between *Q. aliena* and *Q. dentata* was in the patterns of expansion and contraction from the base to the apex of the leaf (Figure 4a). *Q. dentata* leaves exhibited shorter petiole (distance between LM1 and LM2), wider blade tip (distance between LM5 and LM10), deeper lobes (distance between LM7 and LM12), and relatively narrower basal region (distance between LM8 and LM13) than *Q. aliena* leaves along the CV1 axis (Figure 4a).

The DA results also revealed significant morphological differences between *Q. aliena* and *Q. dentata*, largely concordant with the CVA results (Figure 4b, Table 1, *T* ^2^ = 2432; *p* < .0001). DA achieved a high discriminant rate of 98.3% between *Q. aliena* and *Q. dentata* (Figure 4b, Table 1). Furthermore, in pairwise comparisons, 99% and 98% of *Q. aliena* and *Q. dentata* (Figure 4b), 89% and 71% of *Q. aliena* and admixture (Figure 4c), and 86% and 57% of *Q. dentata* and admixture (Figure 4d) were correctly identified, respectively.

**TABLE 1:** The discriminant rate of geometric morphometric method (GMM) on *Q. aliena, Q. dentata*, and admixture based on the data of cross‐validation in discriminant analysis (DA).

Category	Proportion of discriminant (%)	Discriminant rate (%)	Procrustes distance	T‐square	*p*‐value
*Q. aliena*	98.8	98.3	0.12	2432.8	<.0001
*Q. dentata*	97.9				
*Q. aliena*	89.4	79.3	0.07	243.9	<.0001
Admixture	70.5				
*Q. dentata*	86.4	74.3	0.05	183.0	<.0001
Admixture	57.1				

### 3.3 Accuracy of deep learning discrimination

**FIGURE 5:**
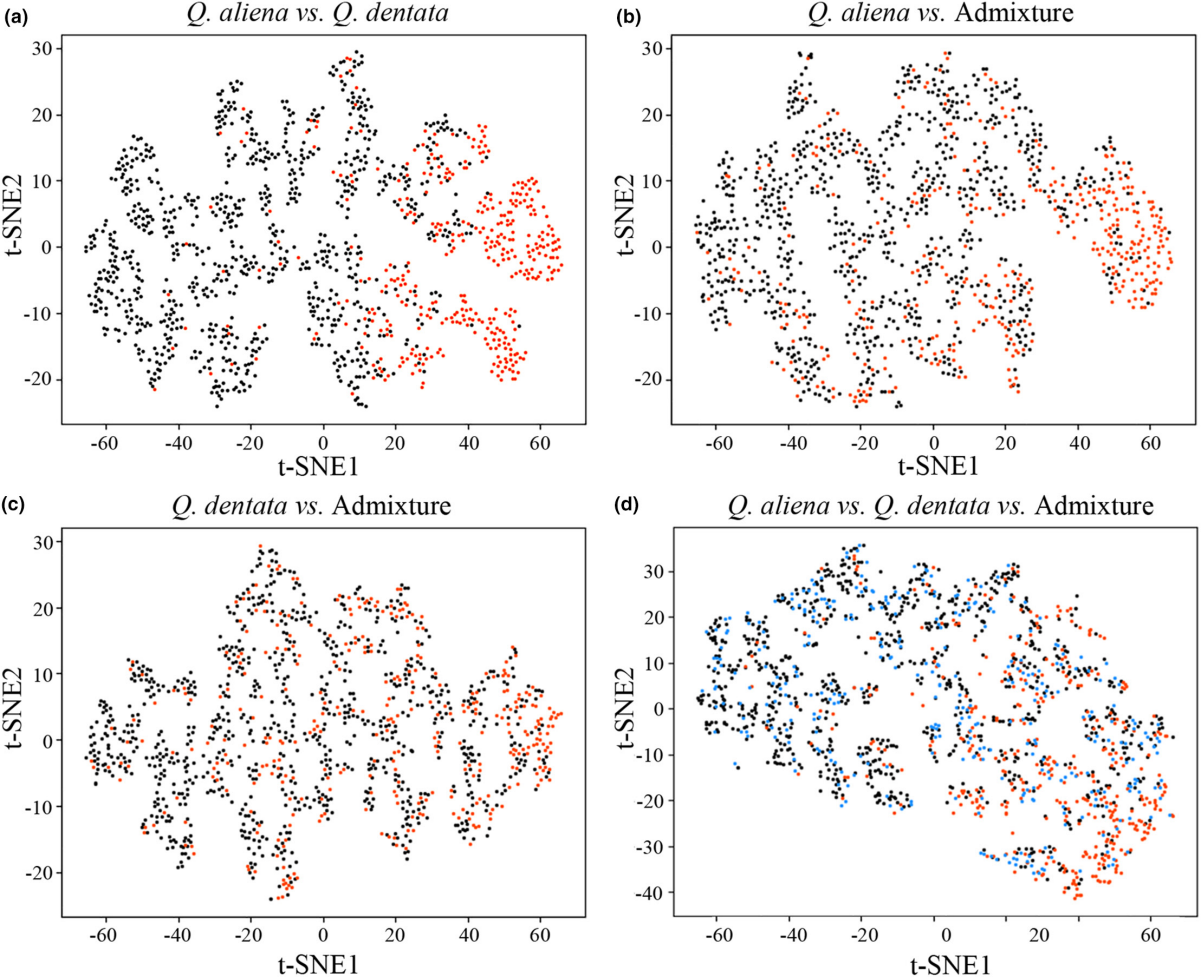
Last layer feature mapping obtained by t‐distributed Stochastic Neighbor Embedding (t‐SNE) for different classifications of (a) *Quercus aliena* vs. *Quercus dentata*, (b) *Quercus aliena* vs. admixture, (c) *Quercus dentata* vs. admixture and (d) *Quercus aliena* vs. *Quercus dentata* vs. admixture.

We trained and evaluated four data sets for *Q. aliena*, *Q. dentata*, and admixture based on the Xception architecture. The test accuracy exhibited rapid improvement from the initial epoch, stabilizing after 20 epochs for the data sets of *Q. aliena* vs. *Q. dentata* and *Q. aliena* vs. *Q. dentata* vs. admixture (Figure S3a,d). Extracting features from the leaf images revealed strong aggregation characteristics within images of the same species, enabling accurate species identification for the *Q. aliena* and *Q. dentata* data sets (Figure 5). The Xception architecture trained on images of *Q. aliena* and *Q. dentata* performed well, with an accuracy rate of 95.8% and an *F*‐score of 0.9 (Table 2). However, the results showed that the recognition accuracy was the lowest for the data set of *Q. dentata*, *Q. aliena*, and admixture (accuracy: 44.5%, *F*‐score: 0.3). The discrimination between *Q. aliena* and the admixture exhibited a higher accuracy rate than *Q. dentata* and the admixture, suggesting that the admixture displayed greater morphological similarity to *Q. dentata* (accuracy: 71.8% vs. 67.9%, Table 1).

**TABLE 2:** Classifier performance for test different groups with Xception model.

Group	Precision	Recall	Accuracy (%)	*F*‐score
*Q. aliena* vs. *Q. dentata*	0.9	0.9	95.8	0.9
*Q. aliena* vs. Admixture	0.8	0.6	71.8	0.7
*Q. dentata* vs. Admixture	0.8	0.5	67.9	0.6
*Q. aliena* vs. *Q. dentata* vs. Admixture	0.3	0.3	44.5	0.3

### 3.4 Cost comparison of species identification

**FIGURE 6:**
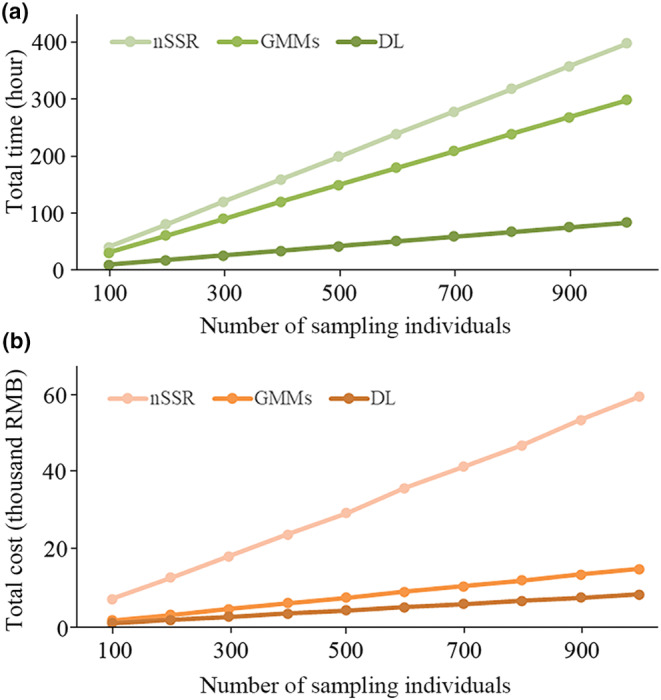
Estimation of (a) time required and (b) cost‐effectiveness for species identification using genotyping (nSSR), geometric morphometric method (GMM), and deep learning (DL) with the number of sampling individuals increases.

A cost analysis comparing the three different methods for species identification revealed that as the number of sampled individuals increased, the total time and cost of nSSR exceeded those of geometric morphology and deep learning methods (Figure 6a,b).

## 4 DISCUSSION

In this study, we conducted a comparative analysis to evaluate the accuracy and efficiency of the geometric morphology and deep learning methods in discriminating closely related oaks, using genetics as a priori classification. Our analysis focused on leaf shape variation, which is related to the physiological characteristics of the species. Considering the intuitive shape of plant species, GMM allows leaf shape variation to be more visualized than traditional morphological measurements. In addition, we used deep learning, an artificial intelligence approach with the ability to process large and complex datasets, to discriminate oak species and highlight the role of the potential of artificial intelligence techniques in species identification.

### 4.1 Species identification using genetic assignment as a priori

Inference regarding species identification based on genetic data alone is likely inadequate, and species identification should be conducted considering morphology (Carstens et al., 2013). There are two approaches to this integration. One is the morphological differences serve as the basis for taxonomic hypotheses that are validated using genetic data (e.g., Gugerli et al., 2007; Welton et al., 2013). The other is interpreted as morphologic variation in the context of identification from genetic data as a robust methodology to support the results of species identification (e.g., Liu et al., 2018; Stech et al., 2013). In our study, we used the latter approach for species identification of the two oaks using genetic analysis as a priori. Using the Bayesian approach, we successfully assigned over 90% of the samples to one of two distinct clusters corresponding to the previously described species, making it possible to estimate the genetic structure of each species and identify cases of introgression.

If no single morphological recognition trait exists in two related species, multivariate analyses are ideal tools to identify parameters that differentiate between groups of individuals (Rellstab et al., 2016). The results of CVA indicated that there was some variation in the leaf shape of *Q. aliena* and *Q. dentata*, with admixture individuals exhibiting intermediate leaf shapes. Notably, the difference in leaf shape between *Q. aliena* and *Q. dentata* was mainly concentrated in the leaf tip and base according to the transformation grid. When the admixture was influenced by both parent species, their leaf shape represented an average leaf shape of both, but the confidence ellipse overlap was larger and the leaf shape was more similar to that of *Q. dentata*. In DA comparisons, and 71% and 89% of the admixture and *Q. aliena*, 57% and 86% of the admixture and *Q. dentata* trees were correctly classified, respectively, which was consistent with the CVA result that the shape of admixture individuals was close to *Q. dentata*. These morphological analyses revealed significant differences between species and highlighted those mixed genotypes (admixture individuals) are a mosaic of phenotypes with intermediate characteristics of the parental species (Gugerli et al., 2007; Viscosi et al., 2009).

We also used deep learning, a convolutional neural network that automatically extracts image features without manual intervention, to extract the leaf features. This approach overcame the limitations of traditional plant leaf recognition that relied on manpower based on the Xception architecture with excellent classification accuracy and good generalization ability. Our deep learning analysis, used high‐resolution scanned leaf images with a uniformly white background, which was not a photograph taken in the habitat, minimizing errors introduced by the machine learning algorithm. In the deep learning analysis, a higher mean discrimination was observed between *Q. aliena* and *Q. dentata* with a higher detection index accuracy. When the admixture individuals were considered as a separate taxon and verified by three classifications, the accuracy index was 45%, consistent with the GMM result indicating that admixture individuals could not be accurately identified because of their intermediate form. Interestingly, the resolution between admixture individuals and *Q. dentata* was lower, supporting the finding that the leaf shape of admixture individuals was more similar to that of *Q. dentata*, which is consistent with the results of CVA in GMM. These findings suggest that oaks retain high levels of fitness variation, with *Q. aliena* being more favored by the selection of leaf morphological traits.

### 4.2 Comprehensive comparison of different identification methods

When comprehensively comparing the different methods for plant species identification, it is sensible to consider the overall classification accuracy of the results as well as the accuracy and efficiency of the resources. Using a variety of approaches, we found that each has its own advantages and limitations in plant species identification. We used the molecular level as the criterion to identify the two oak species and their admixture, and subsequently combined GMM and deep learning methods to analyze the identification of *Q. aliena*, *Q. dentata*, and admixture. The identification results of nSSR provide a genetic basis. However, nSSRs require a large number of molecular experiments making it time‐consuming and costly.

Plant morphological identification was implemented by qualitative comparison to distinguish differences in leaf traits among species, transforming species image information into data for statistical analysis. We acquired a large amount of leaf shape information from the images, simplifying the process of morphological data collection compared to traditional morphological measurements. GMM can digitize phenotypic characteristics based on manually extracted landmarks, providing reliable quantitative approaches to leaf shape variation (Viscosi et al., 2011). In particular, GMM has demonstrated its utility in studying shape variations and identifying developmental patterns across various landmarks in individuals, using tools such as morphoj and the R package shapes (Chitwood et al., 2016; Viscosi, 2015). However, GMM requires strong subjective judgment in the manual marking of the feature selection.

Deep learning has been successfully used to identify related species, providing valuable insights for the identification of other species (Işık et al., 2021). Deep learning eliminates the manual search for suitable characteristics by automatically learning relevant characteristics, shortening the classification time, and improving the discrimination accuracy for this specific application. Compared with GMM, deep learning shows advantages in accuracy, as well as significant advantages in terms of cost and time, and in large samples. However, deep learning method requires a large number of basic images to distinguish individuals with similar phenotypes. The results from GMM and deep learning indicated that leaf shape analysis can effectively distinguish the two species. Deep learning is particularly advantageous in inter‐species identification, while GMM is better at identifying mixed individuals and pure individuals, as well as visualizing the shape difference. Deep learning showed a greater advantage in species identification because feature extraction in deep learning contains more than 13 landmarks in GMM. Feature extraction of leaves can be further enhanced to improve the accuracy of identification, and the overall leaf morphology can be clearly presented by enhancing the leaf vein characteristics based on the original data (Grinblat et al., 2016).

In this study, we achieved favorable identification results between two related species using deep learning. However, complicated as oaks further verification is required to determine whether the deep learning model can be applied for their identification. The leaf shape was the only characteristic used in the classifiers in our study. Given the degree of leaf morphology similarity, and incorporating additional features such as flower, vein, or pollen microscopic imaging into the classifier, better performance of species identification might be expected.

## AUTHOR CONTRIBUTIONS

**Min Qi:** Conceptualization (supporting); data curation (lead); formal analysis (lead); investigation (lead); project administration (supporting); validation (lead); visualization (equal); writing – original draft (lead); writing – review and editing (equal). **Fang Du:** Conceptualization (lead); funding acquisition (lead); investigation (supporting); project administration (lead); resources (lead); supervision (lead); validation (lead); visualization (equal); writing – original draft (supporting); writing – review and editing (lead). **Fei Guo:** Formal analysis (equal); methodology (equal); software (lead); validation (equal); visualization (equal); writing – review and editing (equal). **Kangquan Yin:** Conceptualization (equal); funding acquisition (lead); validation (supporting); visualization (equal); writing – review and editing (equal). **Jijun Tang:** Methodology (equal); software (equal); writing – review and editing (equal).

## FUNDING INFORMATION

This research was supported by a grant from Fundamental Research Funds for the Central Universities (2021ZY80) to KY, Science and Technology Innovation of Inner Mongolia Autonomous Region (2022JBGS0020) to KY, a grant from National Forestry and Grassland Administration of China (No. KJZXSA202212) to FD and the National Natural Science Foundation of China (No. 42071060) to FD.

## CONFLICT OF INTEREST STATEMENT

The authors declare no competing interests.

## Supporting information


Appendix S1

## Data Availability

Genotyping data can be found at https://doi.org/10.6084/m9.figshare.24346693.v1, and leaf morphological data can be obtained at https://doi.org/10.6084/m9.figshare.24346705.v1. Photographs of sampling sites can be obtained at https://www.oakofchina.org/photo‐of‐sampling/.
