# Supplementary material for: Species identification through deep learning and geometrical morphology in oaks (Quercus spp.): Pros and cons
Source: Ecol Evol. 2024 Feb 13;14(2):e11032. doi: 10.1002/ece3.11032 (PMC10864717; doi:10.1002/ece3.11032)
Supplement: Supplementary file 1 — Appendix S1 [file ECE3-14-e11032-s001.docx]

**SUPPORTING INFORMATION**

**Figure S1**. Geographic distribution of the sampled populations for *Quercus aliena* and *Quercus dentata*. Open circles: *Quercus aliena*, filled black circles: *Quercus dentata.* For the details of sampling locations see supporting Table S1.

**Figure S2**. Population clusters identified with Structure software. (a) Variation of delta K as a function of K, calculated over ten replicates. (b) Log-likelihood value of data, Ln P(K), as a function of K for ten replicates.

**Figure S3**. Classification accuracy as a function of epoch using deep learning for different classification of (a) *Quercus aliena* vs. *Quercus dentata*, (b) *Quercus aliena* vs. admixture, (c) *Quercus dentata* vs. admixture and (d) *Quercus aliena* vs. *Quercus dentata* vs. admixture.

**Figure S1**


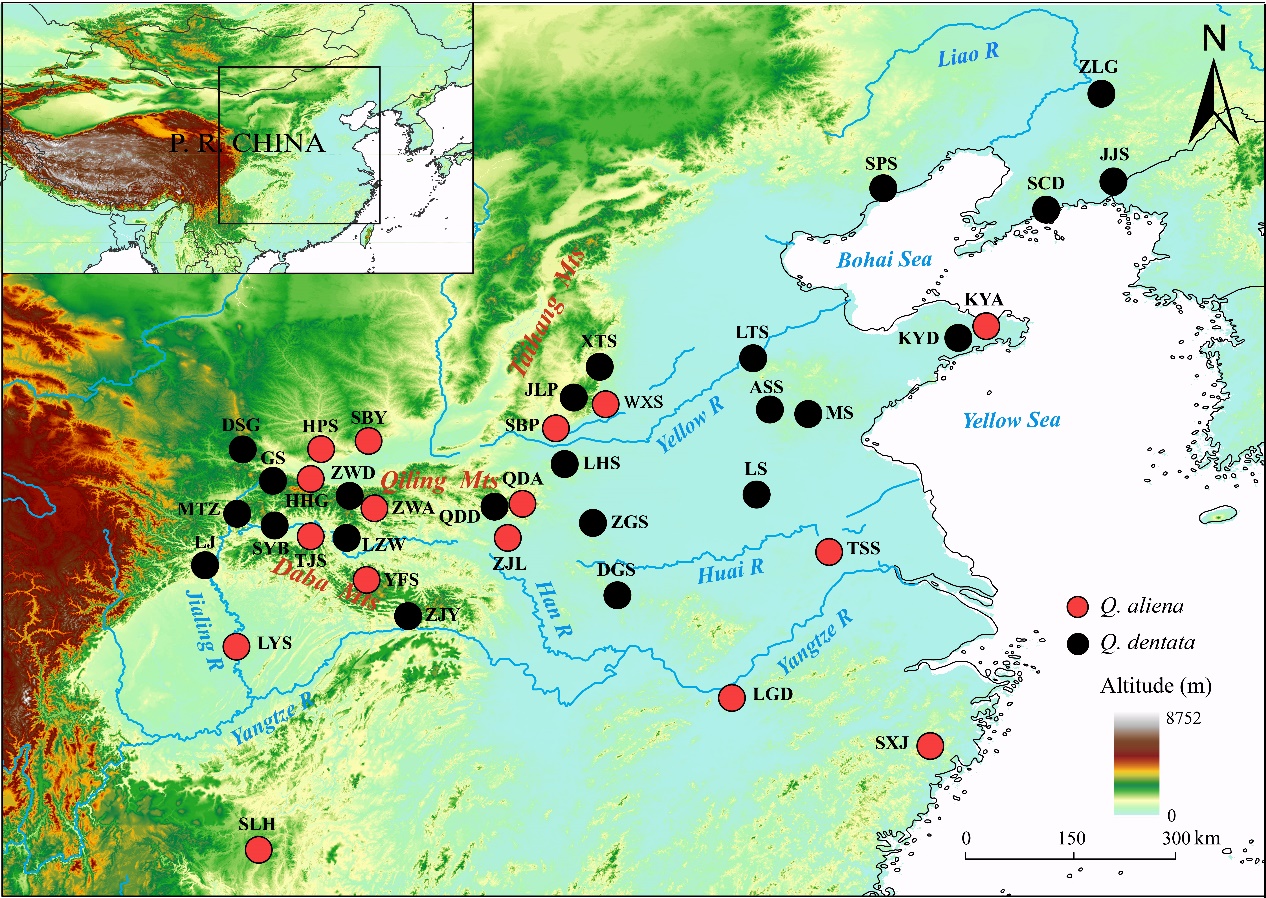


**Figure S2**


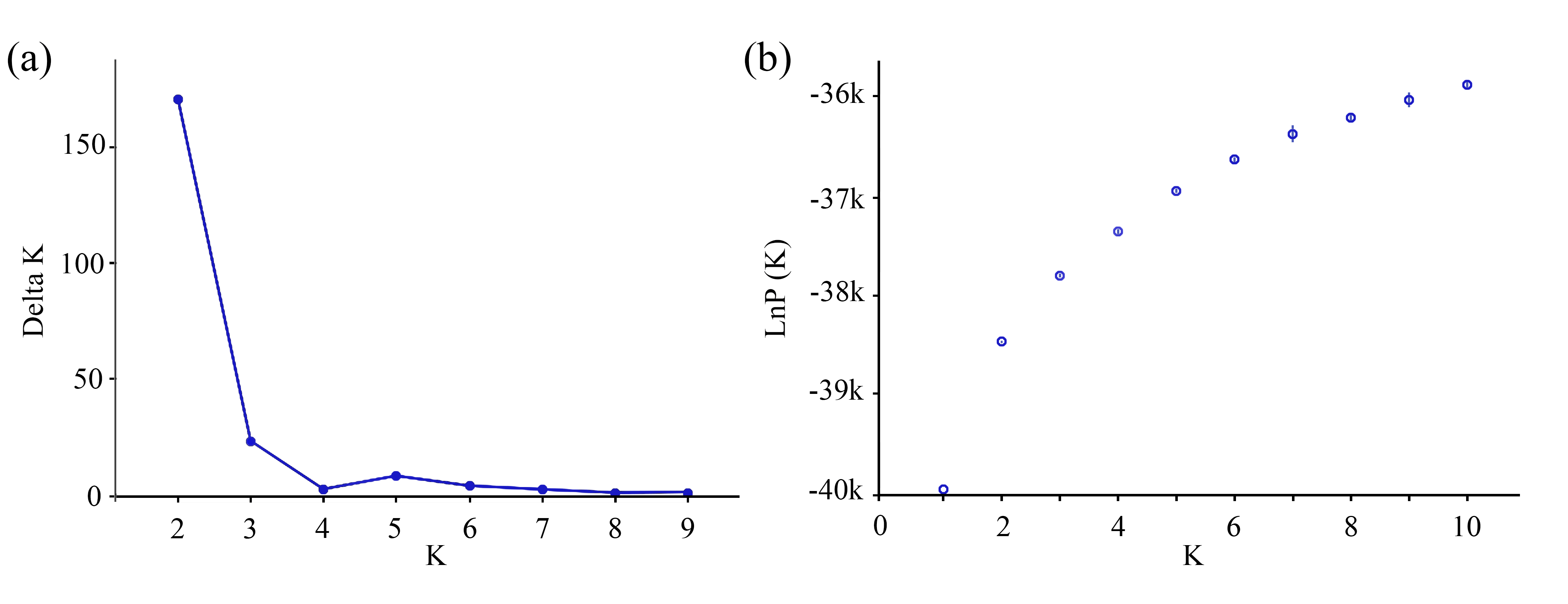


**Figure S3**

**
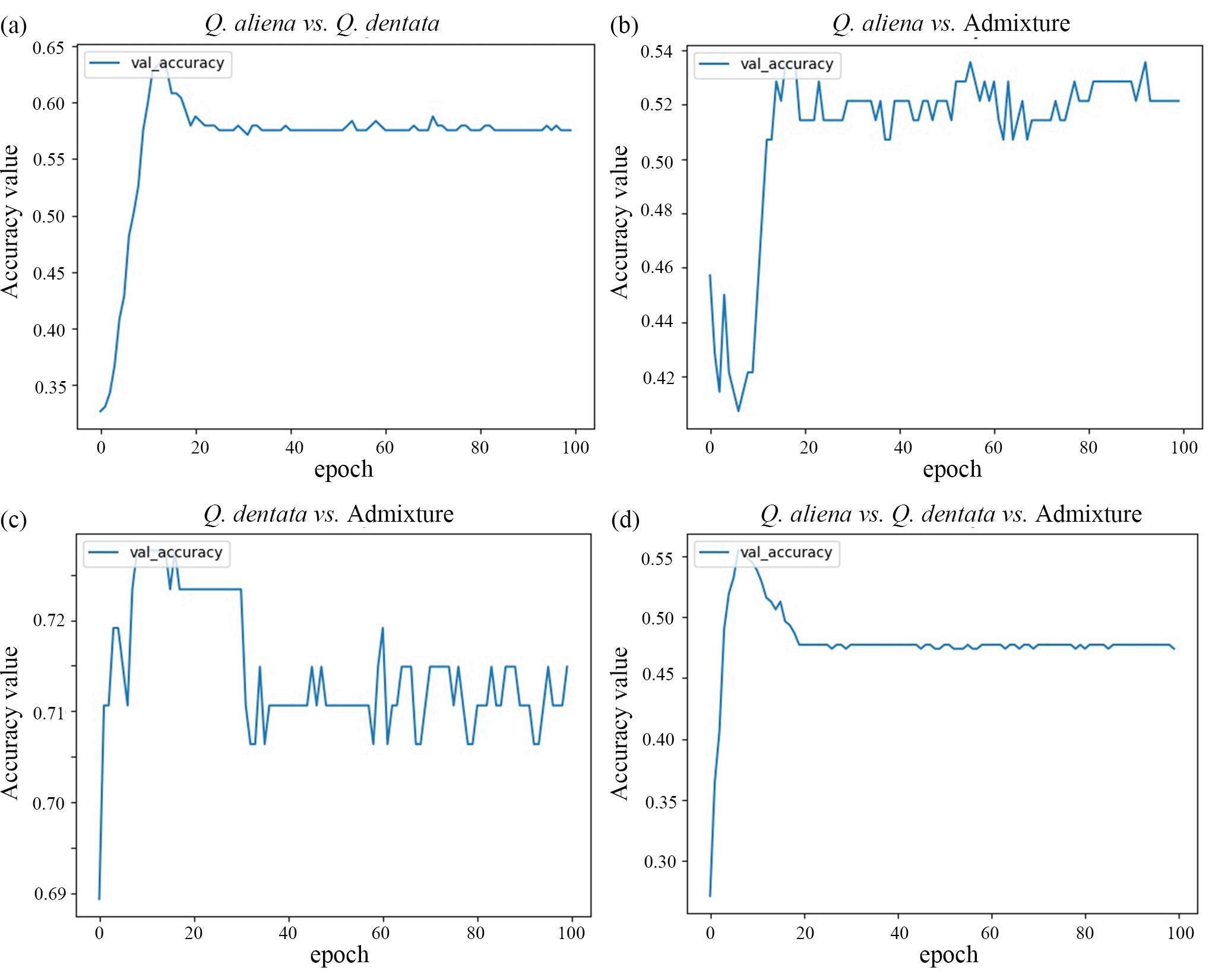
**

**Table S1** Details of the 39 natural populations of *Q. aliena* and *Q. dentata*.

| Code | Sampling location | Latitude(N) | Longitude(E) | Altitude(m) | Individual number | Leaf number |
| --- | --- | --- | --- | --- | --- | --- |
| ***Q. aliena*** |  |  |  |  |  |  |
| HPS | Haoping Temple, Meixian County, Shaanxi Province | 34.09 | 107.71 | 1130 | 23 | 50 |
| KYA | Kunyu Mountain, Yantai, Shandong Province | 37.28 | 121.64 | 169 | 12 | 60 |
| LGD | Longgong cave, Jiujiang City, Jiangxi Province | 29.7 | 116.47 | 130 | 6 | 29 |
| LYS | Lingyun Mountain, Nanchong City, Sichuan Province | 30.75 | 106.2 | 520 | 4 | 20 |
| QDA | Qiaoduan Town, Nanyang City, Henan Province | 33.55 | 112.08 | 456 | 17 | 75 |
| SBP | Shibanpo, Macaque Nature Reserve, Henan Province | 35.22 | 112.83 | 900 | 18 | 50 |
| SBY | Shibianyu, Xi'an City, Shaanxi Province | 35.02 | 108.96 | 910 | 9 | 45 |
| SLH | Shili River Beach, Guiyang City, Guizhou Province | 26.45 | 106.68 | 1130 | 5 | 20 |
| SXJ | Shenxianju, Taizhoun City, Zhejiang Province | 28.7 | 120.61 | 180 | 10 | 48 |
| TJS | Tiejiashu Scenic Spot, Xi'an City, Shaanxi Province | 33.87 | 107.76 | 1580 | 9 | 45 |
| TSS | Tieshan Temple, Huaian City, Jiangsu Province | 32.73 | 118.48 | 99 | 12 | 59 |
| WXS | Wanxian Mountain, Xinxiang City, Henan Province | 35.73 | 113.6 | 960 | 10 | 50 |
| YFS | Yaofu Mountain, Pingli County, Shaanxi Province | 32.13 | 108.87 | 1400 | 5 | 25 |
| ZWA | Ziwuyu, Xi'an City, Shaanxi Province | 33.56 | 108.55 | 1132 | 12 | 60 |
| HHG | Honghe valley, Meixian County, Shaanxi Province | 34.06 | 107.75 | 1307 | 9 | 45 |
| ZJL | Baotianman Nature Reserven, Nanyang, Henan Province | 33.06 | 111.84 | 1020 | 10 | 23 |
| ***Q. dentata*** | |  |  |  |  |  |
| ASS | Anshan Temple, Jining City, Shandong Province | 35.56 | 117.38 | 240 | 21 | 100 |
| DGS | Dagui Temple, Guangshui City, Hubei Province | 31.82 | 114.08 | 270 | 7 | 35 |
| DSG | Dasanguan in Baoji City, Shaanxi Province | 34.27 | 107.01 | 890 | 23 | 100 |
| GS | Guanshan, Taibai County, Shaanxi Province | 34.04 | 107.24 | 1470 | 11 | 50 |
| JJS | Jinjiangshan Park, Dandong City, Liaoning Province | 40.13 | 124.36 | 80 | 11 | 54 |
| JLP | Jinlingpo Village, Jiaozuo City, Henan Province | 35.71 | 113.33 | 590 | 24 | 99 |
| KYD | Kunyu Mountain, Yantai, Shandong Province | 37.28 | 121.64 | 169 | 20 | 100 |
| LHS | Lotus Temple, Dengfeng Forest Farm, Henan Province | 34.46 | 112.95 | 640 | 15 | 50 |
| LS | Longji Mountain, Huaibei City, Anhui Province | 33.9 | 116.97 | 180 | 12 | 50 |
| LJ | Luojiashan, Dazhou City, Sichuan Province | 32.45 | 105.58 | 980 | 14 | 69 |
| LTS | Liantaishan, Jinan City, Shandong Province | 36.44 | 116.93 | 350 | 14 | 69 |
| LZW | Liangziwan, Ankang city, Shaanxi province | 33.33 | 108.27 | 960 | 5 | 23 |
| MS | Mengshan, Linyi City, Shandong Province | 35.55 | 117.97 | 322 | 20 | 77 |
| MTZ | Miaotaizi, Liuba County, Shaanxi Province | 33.69 | 106.85 | 1220 | 22 | 100 |
| QDD | Qiaoduan Town, Nanyang City, Henan Province | 33.55 | 112.08 | 456 | 20 | 100 |
| SCD | Shicheng Island, Dalian City, Liaoning Province | 39.52 | 122.98 | 81 | 16 | 79 |
| SPS | Shangpingshan Village, Hebei Province | 40.07 | 119.52 | 185 | 21 | 100 |
| SYB | Sangyuanba Township, Shaanxi Province | 33.7 | 107.14 | 1020 | 21 | 100 |
| XTS | Xiantai Mountain, Anyang City, Henan Province | 36.17 | 113.75 | 590 | 16 | 49 |
| ZGS | Zhigushan, Pingdingshan City, Henan Province | 33.32 | 113.51 | 210 | 20 | 50 |
| ZJY | Zhangjiayazi, Wuxi County, Chongqing | 31.4 | 109.77 | 1410 | 8 | 40 |
| ZLG | Zhonglougou, Tieling City, Liaoning Province | 42.06 | 124.03 | 280 | 10 | 50 |
| ZWD | Ziwuyu, Xi'an City, Shaanxi Province | 33.56 | 108.55 | 1132 | 16 | 80 |
| **Total** |  |  |  |  | **538** | **2328** |

**Table S2** Detailed information of 12 pairs of SSR primers.

| SSR Locus | Tm（°C） | Motif | Primer Sequence（5'-3'） | Allele size rang（bp） | Reference |
| --- | --- | --- | --- | --- | --- |
| GOT021 | 56 | AT | AGAAAGTTCCAGGGAAAGCA | 111-128 | Durand *et al*., 2010 |
|  |  |  | CTTCGTCCCCAGTTGAATGT |  |  |
| FIR026 | 56 | TC | CTTCATGCACCAATTCCTCA | 208-217 | Durand *et al*., 2010 |
|  |  |  | GGCCATGTATGTGTGCAAAA |  |  |
| QmC00716 | 56 | TC | AAGAGAACCCATTCCATCCCTGA | 261-287 | Ueno *et al*., 2008 |
|  |  |  | GTTTCCCGAACAGTGGTTTCTTGA |  |  |
| POR017 | 54 | CT | CCCATATCCCTCTACGAAAGAA | 140-169 | Durand *et al*., 2010 |
|  |  |  | CTGGAGATGACATAGTGTCTCAAA |  |  |
| FIR015 | 56 | AC | ACCCTAAAACCCCAATCACC | 128-138 | Durand *et al*., 2010 |
|  |  |  | CGGATCTTCGGCTATTCTTG |  |  |
| QmC00932 | 54 | TC | AGGCTCAAAACAAAACCAAACCG | 247-260 | Ueno *et al*., 2008 |
|  |  |  | GTTTCCCCTTTCCCATAATCAAACCCT |  |  |
| DN950446 | 56 | AG | TCTCTTTCTCCGTCCATTATCGC | 155-185 | Ueno & Tsumura, 2008 |
|  |  |  | GTTTCTCCACAGACCCCATTTCC |  |  |
| WAG068 | 56 | AG | TCTGCAACAAAACCAAAACAC | 165-195 | Durand *et al*., 2010 |
|  |  |  | CGGAGGAGAGAGTCAGCAAC |  |  |
| PIE271 | 56 | TC | CACACTCACCAACCCTACCC | 197-247 | Durand *et al.,* 2010 |
|  |  |  | GTGCGGTTGTAGACGGAGAT |  |  |
| QmC02052 | 56 | AG | CACACCCAGATCCACAAAACTCC | 250-300 | Ueno *et al*., 2008 |
|  |  |  | GTTTGCCTCTACGGTCTCCCTCTT |  |  |
| GOT011 | 56 | TC | CCCCACCGTCTACTCTCAAA | 197-255 | Durand *et al*., 2010 |
|  |  |  | GCGTTCACCACGTCCATAAT |  |  |
| WAG066 | 56 | AG | AACCTGTTTGGCTTCGTGTG | 128-244 | Durand *et al*., 2010 |
|  |  |  | AACAAAAGATTGGGAGGTGC |  |  |

**Cited references:**

Durand, J., Bodénès, C., Chancerel, E., Frigerio1, J., Vendramin, G., Sebastiani, F., …Alberto1, F., Dumoulin1, P., Guichoux1, E., Daruvar, A., Kremer, A., & Plomion, C. (2010). A fast and cost-eﬀective approach to develop and map EST-SSR markers: oak as a case study. *BMC Genomics*, 11, 570. <https://doi.org/10.1186/1471-2164-11-570>

Ueno, S., & Tsumura, Y. (2008). Development of ten microsatellite markers for *Quercus mongolica* var. *crispula* by database mining. *Conservation Genetics*, 9(4), 1083-1085. <https://doi.org/10.1007/s10592-007-9462-4>

Ueno, S., Taguchi, Y., & Tsumura, Y. (2008). Microsatellite markers derived from *Quercus mongolica* var. *crispula (Fagaceae)* inner bark expressed sequence tags. *Genes & Genetic Systems*, 83(2), 179-87. [https://doi.org/ 10.1266/ggs.83.179](%20https:/doi.org/%2010.1266/ggs.83.179)

**Table S3** The description of 13 landmarks location.

| Landmark | Description |
| --- | --- |
| LM1 | Junction of the petiole and branch |
| LM2 | Junction of the blade and petiole |
| LM3 | Apex of the leaf |
| LM4 | The first sinus of the right-hand leaf apex |
| LM5 | The first lobe immediately above the apex of the right-hand leaf apex |
| LM6 | Tip of the lobe at the largest width of the right-hand leaf |
| LM7 | The sinus immediately above the lobes of 6 |
| LM8 | The first basal lobe of the right-hand leaf starting from the petiole |
| LM9 | The first sinus of the left-hand leaf apex |
| LM10 | The first lobe immediately above the apex of the left-hand leaf apex |
| LM11 | Tip of the lobe at the largest width of the left-hand leaf |
| LM12 | The sinus immediately above the lobesof 11 |
| LM13 | The first basal lobe of the left-hand leaf starting from the petiole |
